# Curcumin inhibits angiogenesis and improves defective hematopoiesis induced by tumor-derived VEGF in tumor model through modulating VEGF-VEGFR2 signaling pathway

**DOI:** 10.18632/oncotarget.3625

**Published:** 2015-05-08

**Authors:** Zhongping Fu, Xiao Chen, Shengwen Guan, Yanju Yan, Huan Lin, Zi-Chun Hua

**Affiliations:** ^1^ State Key Laboratory of Quality Research in Chinese Medicines, Macau University of Science and Technology, Avenida Wai Long, Taipa, Macau, China; ^2^ The State Key Laboratory of Pharmaceutical Biotechnology, School of Life Sciences, Nanjing University, Nanjing, China; ^3^ Nanjing Industrial Innovation Center for Pharmaceutical Biotechnology, Nanjing, Jiangsu, China; ^4^ Changzhou High-Tech Research Institute of Nanjing University and Targetpharma Laboratory, Changzhou, Jiangsu, China; ^5^ Jiangsu Simcere Pharmaceutical R&D Co., Ltd., Nanjing, China; ^6^ College of Pharmacy, Nanjing University of Chinese Medicine, Nanjing, Jiangsu, China

**Keywords:** curcumin, angiogenesis, VEGF, anemia, extramedullary hematopoiesis

## Abstract

Curcumin, a natural polyphenol compound from the perennial herb *Curcuma longa*, has been proved to be beneficial for tumor-bearing animals through inhibiting tumor neovasculature formation, but the underlying mechanisms are unclear. Here, we aim to test whether curcumin affects VEGF-VEGFR2 signaling pathway and attenuates defective hematopoiesis induced by VEGF in tumor model. We demonstrated that curcumin inhibited proliferation, migration of HUVEC under VEGF stimulation and caused HUVEC apoptosis, and blocked VEGFR2 activation and its downstream signaling pathways *in vitro*. Furthermore, in VEGF over-expressing tumor model, curcumin significantly inhibited the tumor growth accelerated by VEGF in a dose-dependent manner and improved anemia and extramedullary hematopoiesis in livers and spleens of tumor-bearing mice induced by tumor-derived VEGF. Immunohistochemical analysis showed that curcumin normalized vasculature structures of livers and reduced tumor microvessel density. ELISA revealed that curcumin suppressed VEGF secretion from tumor cells both *in vitro* and *in vivo*. Survival analysis showed that curcumin significantly improved survival ability of VEGF tumor-bearing mice. Taken together, these findings establish curcumin as a modulator of VEGF and VEGF-VEGFR2 signaling pathway, with potential implication for improving the quality of life of cancer patients.

## INTRODUCTION

VEGF, as a mitogen and survival factor for vascular endothelial cells, is a crucial regulator of vascular development during embryogenesis as well as blood-vessel formation in the adult [1]. VEGF acts through two high-affinity receptor tyrosine kinases, VEGFR1/flt-1 and VEGFR2/KDR/flk-1, both are expressed on normal vascular endothelial cells [2]. VEGFR2 is considered to be the predominant mediator of VEGF-stimulated endothelial cell migration, proliferation, survival, and enhanced vascular permeability [3–5]. The tumor growth *in vivo* requires new vessels to provide nutrition and oxygen, thus, the tumor cells recruit proangiogenic cytokines to induce tumor angiogenesis, and VEGF, mainly secreted from tumors, has been demonstrated to be the critical proangiogenic stimulator in neovascularization during the process of tumor development [6–10]. The tumor vasculature is an increasingly attractive target for development of anticancer drugs [11] and antiangiogenic drugs in combination with chemotherapy improve survival in patients with certain types of cancers [12]. Tumor-derived angiogenic factors act locally to promote tumor angiogenesis, but they may also enter the circulation and have an effect on the healthy vasculature [13]. VEGF-VEGFR2 signaling system is essentially required for maintenance of a subset of vasculatures in healthy tissues and organs [14–15]. When optimal expression levels are altered, VEGF often causes pathological disorders by triggering uncontrolled vascular responses that include pathological vasculogenesis, angiogenesis, and tissue edema [16].

Curcumin, a natural polyphenol from turmeric, which presents strong anti-oxidative, anti-inflammatory and anti-septic properties, has been used for centuries to treat inflammation, tumor and other diseases [17]. Accumulating evidences suggest that curcumin shows its anti-tumor activity by modulating various targets either through direct interaction or through modulation of gene expression [18–19]. Curcumin has been demonstrated to possess direct antiangiogenic activity *in vitro* and *in vivo* [20–23]. Curcumin inhibits VEGF-mediated angiogenesis in human intestinal microvascular endothelial cells through COX-2 and MAPK inhibition [24]. Curcumin down-regulates gene expression of VEGF, angiopoietin 1 and 2 in tumor cells and suppresses VEGFR2 expression in HUVEC [25–26]. In addition, curcumin has been found to inhibit VEGF production from various tumor cells though down-regulation of HIF1-α expression [27–28].

Based on the above considerations, the purpose of this present study was to investigate the effect of curcumin on VEGF-VEGFR2 signaling pathway and pathological disorders induced by VEGF. To test this, effect of curcumin was evaluated *in vitro* and in VEGF tumor model. Our results showed that curcumin inhibited VEGF induced HUVEC proliferation and migration and caused apoptosis of HUVEC. And curcumin blocked VEGFR2 mediated signaling pathways through suppressing phosphorylation of VEGFR2 induced by VEGF. Furthermore, curcumin inhibited tumor growth accelerated by VEGF and improved several pathological changes including anemia, hepatosplenomegaly and extramedullary hematopoiesis in livers and spleens of tumor-bearing mice induced by VEGF in tumor model. In addition, curcumin reduced circulating VEGF and prolonged survival times of tumor-bearing mice. Taken together, our data identify curcumin as a blocker of VEGF-VEGFR2 signaling pathways and suggest that curcumin-based therapy has the possibility to improve quality of life and lifespan of cancer patients.

## RESULTS

### Curcumin inhibited VEGF induced HUVEC proliferation and migration *in vitro* and caused apoptosis of HUVEC

**Figure 1:**
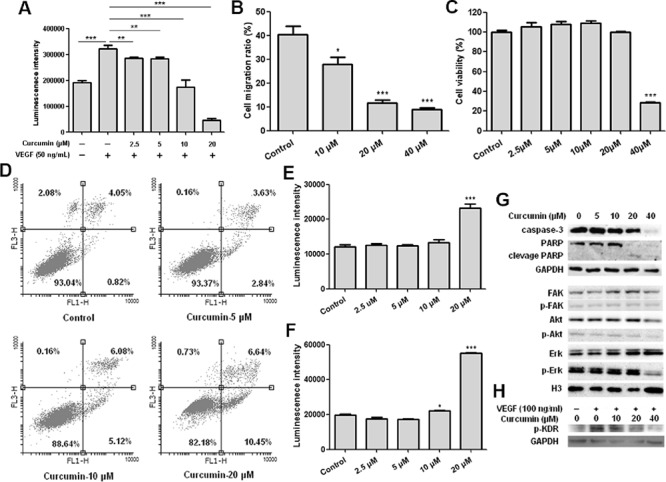
Curcumin inhibited HUVEC proliferation and migration, induced apoptosis of HUVEC and blocked activation of VEGFR2 and VEGFR2 mediated signaling pathways **A.** Results of HUVEC viability assay, HUVEC was treated with curcumin for 72 hours under VEGF stimulation, **p < 0.01, ***p < 0.001; **B.** Results of wound healing assay, HUVEC cell migration ratio after curcumin treatment for 8 hours; **C.** Results of MTT assay, cell viabilities of HUVEC after curcumin treatment for 8 hours; **D.** HUVEC cells were collected and stained with annexin V-FITC and PI after treated with curcumin for 24 hours, then determined by flow cytometry; **E.** HUVEC cells were treated with curcumin for 12 hours, then caspase 3/7 activities were determined by Caspase-Glo^®^ 3/7 assay; **F.** HUVEC cells were treated with curcumin for 24 hours, then caspase 3/7 activities were determined by Caspase-Glo^®^ 3/7 assay; **G.** HUVEC cells were treated with curcumin for 24 hours, then cell lysates were collected and western blot was conducted for the indicated proteins; **H.** VEGFR2 phosphorylation analysis, PAE-KDR was starved for 16 hours, then treated with curcumin in serum-free medium for 8 hours, stimulated with 100 ng/ml VEGF for 15 minutes, cell lysates were collected and western blot was conducted for phosphorylation of VEGFR2/KDR; (*p < 0.05 vs control; ***p < 0.001 vs control)

Human umbilical vein endothelial cell (HUVEC) plays a key role in vascular sprout and growth and often be used to evaluate anti-angiogenesis activity *in vitro* [29]. In order to evaluate the effect of curcumin on VEGF-VEGFR signaling pathway *in vitro*, cell viability and migration assays of HUVEC under VEGF stimulation were conducted, our results showed that VEGF significantly stimulated HUVEC proliferation and curcumin efficiently inhibited VEGF induced HUVEC proliferation (Figure 1A). Wound healing assay revealed that migration of HUVEC stimulated by VEGF was significantly suppressed by curcumin without loss of cell viability (Figure 1B, 1C). These results give us the convincing evidence that curcumin affects the signaling pathways of VEGF, resulting in inhibition of proliferation and migration.

Results of HUVEC proliferation assay also indicated that curcumin not only inhibited endothelial cells proliferation, but also caused cells step into death. In order to clarify apoptosis or necrosis, we conducted Annexin V-FITC/PI assay and the results determined by flow cytometry showed that percent of apoptosis cells of HUVEC was increased apparently after treatment with curcumin (Figure 1D), indicating that curcumin has the ability to induce apoptosis of HUVEC. And it was further confirmed by determination of caspase 3/7 activities. The caspase 3/7 activities of HUVEC were significantly increased after treatment with 20 μM Curcumin for 12 hours or 24 hours (Figure 1E, 1F). Western blot analysis of HUVEC after treatment with curcumin showed caspase-3 and PARP were obviously cleaved to active caspase-3 and PARP fragments (Figure 1G), which demonstrates that curcumin induces HUVEC apoptosis through activating activities of caspase-3.

### Curcumin inhibited activation of VEGFR2 induced by VEGF and blocked VEGFR2 mediated signaling pathways

As we know, VEGF stimulates endothelial cells proliferation, migration and differentiation through activating VEGF receptors mediated signaling pathways, and VEGFR2 mediated signaling pathways play a key role in angiogenesis, lymphangiogenesis, and vasculogenesis [30–31]. It was reasonable for us to investigate the effect of curcumin on activation of VEGFR2 and its downstream signaling pathways. Porcine aortic endothelial cell line over-expressing human VEGFR2 (PAE-KDR) was used to evaluate the effect of curcumin on phosphorylation of VEGFR2 (KDR), and the results showed that curcumin obviously suppressed phosphorylation of VEGFR2 (Figure 1H), which indicates that curcumin can inhibit the activation of VEGFR2 and block VEGFR2 mediated signaling pathways, and it was confirmed by western blot analysis for HUVEC. After treated with curcumin for 24 hours, phosphorylation of Akt and Erk were observed to be decreased apparently (Figure 1G), since Erk and Akt are key downstream factors of VEGFR2-mediated signaling pathways correlated to proliferation and survival of HUVEC respectively, we confirmed that curcumin blocked VEGFR2 mediated downstream signaling pathways. In addition, RT-PCR analysis of HUVEC after treated with curcumin for 24 hours showed that expression of VEGFR1 and VEGFR2 were decreased obviously, while the expression of HIF-1α and VEGF did not change (data not shown). Together of all, curcumin inhibits proliferation and migration of endothelial cells through suppressing activation and expression of VEGF receptors and blocking VEGF receptors mediated signaling pathways.

### Curcumin inhibited T241-VEGF proliferation *in vitro* and suppressed tumor growth accelerated by tumor-derived VEGF in T241-VEGF tumor model

**Figure 2:**
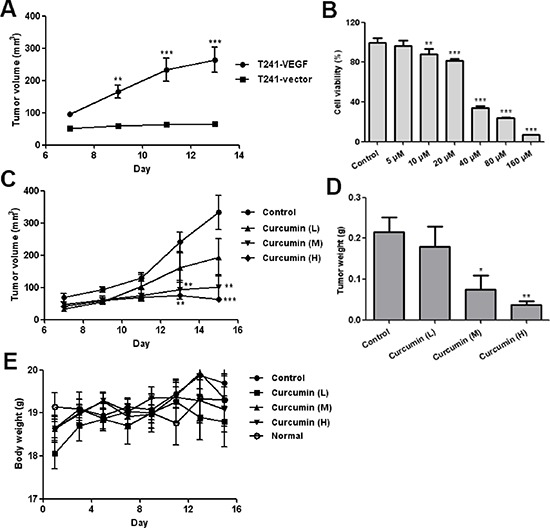
Curcumin inhibited VEGF tumor cells proliferation *in vitro* and tumor growth *in vivo* **A.** Tumor growth curve for T241-vector and T241-VEGF bearing mice; **B.** Result of MTT assay, T241-VEGF was treated with various doses of curcumin for 72 hours; **C.** Tumor growth curve of T241-VEGF bearing mice treated with vehicle and curcumin. L, low dose of curcumin; M, median dose of curcumin; H, high dose of curcumin; **D.** Tumor tissues of various groups were collected and weighed at day 15 after 2 weeks treatment; **E.** Body weights of various groups during treatment. (*p < 0.05 vs control; **p < 0.01 vs control; ***p < 0.001 vs control)

In order to evaluate the effect of curcumin on the VEGF-VEGFR signaling pathway *in vivo*, we used a special tumor cell line T241-VEGF, a mouse fibrosarcoma tumor cell line over-expressing human VEGF. In our xenograft mouse model, we observed that the mouse fibrosarcoma tumor grew significantly faster in the T241-VEGF tumor-bearing mice than that of the T241-vector tumor model (Figure 2A), which confirms the viewpoint that VEGF accelerates tumor growth *in vivo* [30]. The *in vitro* cell viability assay showed that curcumin significantly inhibited the proliferation of T241-VEGF, and the IC_50_ was 31.97 μM (Figure 2B). *In vivo*, curcumin significantly suppressed the tumor growth in T241-VEGF tumor-bearing mice compared with vehicle control in a dose dependent manner (Figure 2C). The tumor weights of the treated groups were decreased significantly (Figure 2D). In contrast, no significant difference of body weights was observed between different groups (Figure 2E), which demonstrates curcumin has the potential against the tumor growth under VEGF stimulation and shows no toxicity to tumor-bearing mice.

### Curcumin improved anemia induced by tumor-derived VEGF in tumor-bearing mice

**Figure 3:**
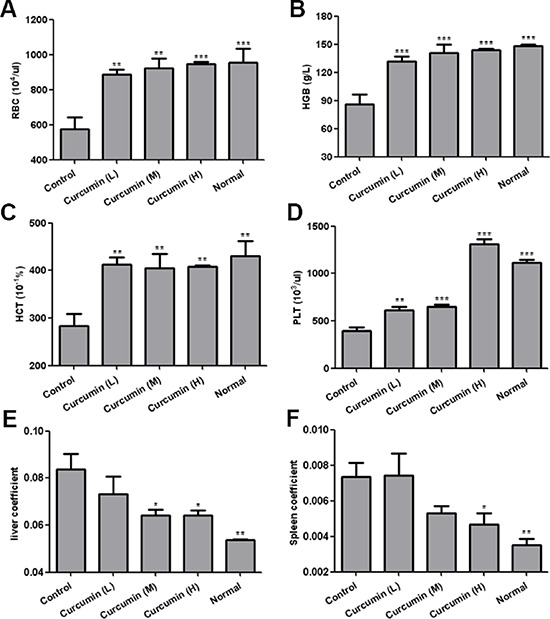
Curcumin improved anemia and hepatosplenomegaly in tumor-bearing mice **A-D.** Peripheral blood was collected by eyeball removal in the presence of anticoagulant EDTA, then analyzed by haematology analyser, red blood cells **A**. hemoglobin **B**. hematocrit **C.** and platelet **D.** were analyzed respectively; **E.** and **F.** Liver tissues and spleen tissues of various groups were collected at day 15 after two-week treatment, liver coefficients **E.** and spleen coefficients **F.** were calculated respectively by tissue weight/body weight. (*p < 0.05 vs control; **p < 0.01 vs control; ***p < 0.001 vs control)

Tumor-derived VEGF induces depletion of hematopoietic cells from bone marrow and results in an anemic symptom in VEGF tumor-bearing mice, and it can be reversed by anti-VEGF or anti-VEGFR2 therapy [32–33]. In our mouse model, we also observed that tumor-bearing mice exhibited severe anemia. Hematological analysis of peripheral blood showed a significant decrease in red blood cells, hemoglobin and hematocrit in T241-VEGF tumor-bearing mice compared with that of normal mice. However, curcumin significantly improved this defective hematopoiesis. The red blood cells, hemoglobin, hematocrit and platelet counts of treated mice were significantly increased compared with that of vehicle control (Figure 3A, 3B, 3C, 3D). These findings reveal that curcumin possesses the same potency as anti-VEGF and anti-VEGFR2 therapy, evidencing that curcumin blocks the function of VEGF-VEGFR2 signaling pathways.

### Curcumin ameliorated extramedullary hematopoiesis in livers and spleens of tumor-bearing mice induced by tumor-derived VEGF

**Figure 4:**
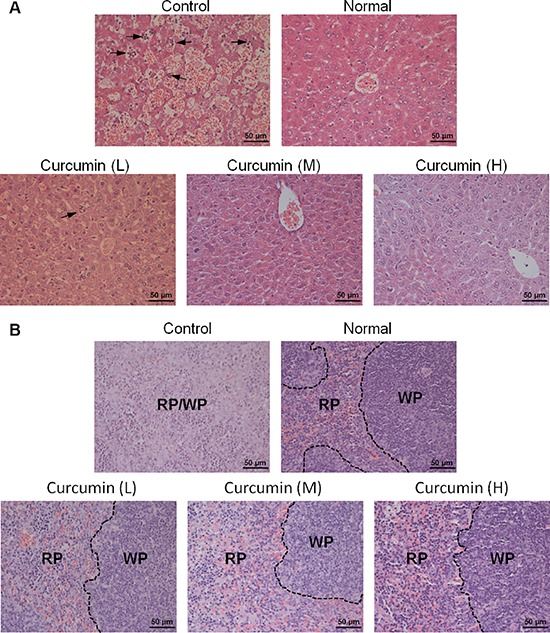
Histological examination of liver tissues and spleen tissues **A.** and **B.** Liver tissues and spleen tissues of various groups were collected, fixed, paraffin embedded, and sectioned after animal sacrifice, then liver sections **A.** and spleen sections **B.** were stained by hematoxylin-eosn (H&E), respectively. Hematopoietic cells islet was labeled with arrow; RP, red pulp, WP, white pulp.

Tumor-derived VEGF acts an endocrine-like hormone to induce hepatomegaly and splenomegaly owing to vessel dilation, tortuosity and activation of hematopoiesis in VEGF tumor-bearing mice, and VEGFR2, not VEGFR1, is the crucial receptor that mediates extramedullary hematopoiesis and tortuosity of vasculatures in these organs [16, 32]. In our study, we also found that livers and spleens of T241-VEGF tumor-bearing mice were significantly different with that of normal mice, the coefficients of livers and spleens of tumor-bearing mice were significantly increased, while liver coefficients and spleen coefficients of curcumin treated groups were significantly decreased, especially for mice treated with high dose of curcumin (Figure 3E, 3F), which suggests that curcumin inhibited hematopoiesis in livers and spleens of tumor-bearing mice induced by tumor-derived VEGF with a dose dependent manner. And it was confirmed by results of histological examination of liver sections and spleen sections. Histological examination of liver sections showed that visible hematopoietic islets were observed in liver sections of VEGF tumor-bearing mice but not in liver sections of normal mice (Figure 4A), which was consistent with previous study [16]. And no hematopoietic islets were found in liver sections of curcumin treated groups, which indicates that curcumin inhibited hematopoiesis in liver tissues of tumor-bearing mice. In addition, histological examination of spleen sections showed that apparent borders between the white pulp (WP) and red pulp (RP) were vanished and replaced by a mixture of WP and RP without distinctive borders in VEGF tumor-bearing mice compared with that of normal mice (Figure 4B), and curcumin apparently protected spleens of tumor-bearing mice from this pathological change. Since active hematopoiesis occurs in red pulp of spleen [16], we confirmed curcumin ameliorates spleen hematopoiesis induced by tumor-derived VEGF. Together of all, these findings demonstrate that curcumin is an efficient blocker of VEGF-VEGFR2 signaling pathways, resulting in ameliorating extramedullary hematopoiesis in livers and spleens of tumor-bearing mice induced by tumor-derived VEGF.

### Curcumin normalized liver vascular structures and decreased tumor microvessel density in tumor-bearing mice

**Figure 5:**
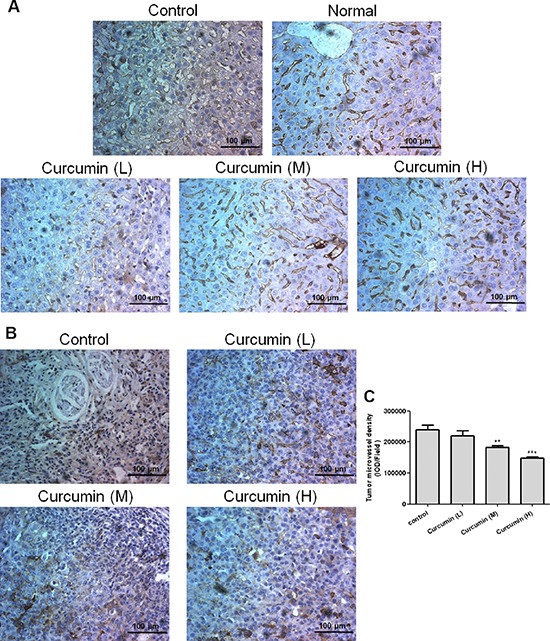
Curcumin normalized vascular structures of livers and decreased tumor microvessel density in tumor-bearing mice **A.** and **B.**, Liver and tumor tissues were collected after animal sacrifice, tissues were fixed, paraffin embedded and sectioned into 4 μm, immunohistochemical analysis was conducted for liver sections **A.** and tumor sections **B.**, respectively; **C.** Quantification of the density of tumor microvessels, the IOD (integrate optical density) was determined by Imagepro Plus, 4–5 pictures for each group were analyzed, field = 346 μm × 435 μm. (**p < 0.01 vs control; ***p < 0.001 vs control)

In order to further validate the mechanism of curcumin on liver hematopoiesis, immunohistochemical analysis was conducted for liver sections and highly dilated sinusoidal hepatic vasculatures were observed in livers of VEGF tumor-bearing mice but not in livers of normal mice, while curcumin obviously reduced vessel dilation and normalized vascular architecture of livers in VEGF tumor-bearing mice (Figure 5A). Since VEGF is known to significantly induce pathological angiogenesis, tortuosity of tumor vasculatures and vasculogenesis in tumors, the effect of curcumin on VEGF tumor angiogenesis was investigated. Immunohistochemical analysis of tumor sections showed that curcumin significantly decreased tumor microvessel density compared to that of the untreated group (Figure 5B, 5C), which indicated curcumin could inhibit tumor angiogenesis stimulated by tumor-derived VEGF. These findings confirm that curcumin inhibits vascular tortuosity and vasculogenesis in tumor and various organs induced by VEGF.

### Curcumin inhibited VEGF production from tumor cells and improved survival ability of tumor-bearing mice

**Figure 6:**
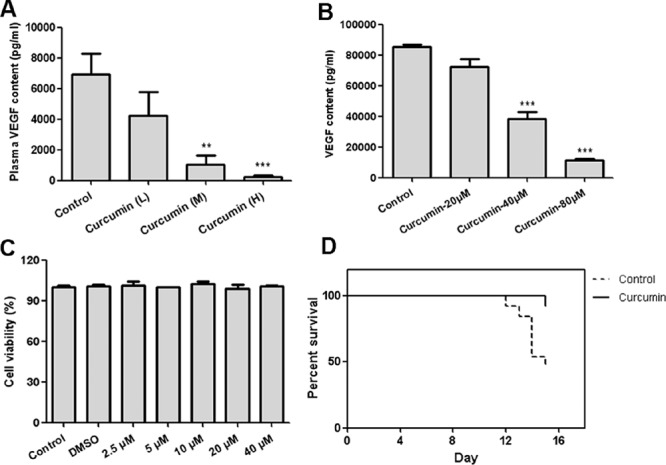
Curcumin decreased VEGF secretion from tumor cells and prolonged survival time of tumor-bearing mice **A.** Plasma was prepared by centrifuging the peripheral blood in the presence of anticoagulant EDTA after animal sacrifice, then VEGF content was determined by ELISA; **B.** Condition medium of T241-VEGF after treated with curcumin for 8 hours was collected and content of VEGF was determined by ELISA; **C.** Cell viability of T241-VEGF after treated with curcumin for 12 hours; **D.** The percentage of survival animals in curcumin treatment group (treated with 5 mM curcumin solution, 100 μL per mouse) and control group is presented during a 14-day-treatment course. (**p < 0.01 vs control; ***p < 0.001 vs control)

Circulating VEGF levels are correlated with severity of cancer associated systemic syndrome (CASS) in preclinical animal models and human cancer patients, high level of VEGF in circulation means more severe pathological disorders [32]. Curcumin improves anemia and extramedullary hematopoiesis induced by tumor-derived VEGF, as tumor secretes VEGF into circulation, we speculated that circulating VEGF of treated mice should be reduced, and it was confirmed by determination of plasma VEGF of tumor-bearing mice. Circulating VEGF of curcumin treated groups was significantly decreased compared with that of control group (Figure 6A). In addition, curcumin was observed to significantly suppress VEGF secretion from T241-VEGF *in vitro* when cell viabilities of T241-VEGF were maintained (Figure 6B, 6C), indicating that curcumin interrupts VEGF production from tumor cells, which was consistent with previous studies [27–28]. Survival time of tumor-bearing animals is impaired by cancer associated systemic syndrome [32, 34]. However, in this VEGF tumor model, curcumin significantly prolonged survival time of tumor-bearing mice compared with vehicle control (Figure 6D), which indicates that curcumin confers survival advantage to tumor-bearing mice by improving pathological changes induced by tumor-derived VEGF.

## DISCUSSION

Curcumin, which is a small molecular weight component of turmeric, has been studied for its wide-ranging effects on tumorigenesis, angiogenesis, apoptosis, and signal transduction pathways [35–36]. Previous studies show that curcumin inhibits angiogenesis through inhibiting VEGF production from tumor cells [23, 27–28, 37] and down-regulating expression of VEGF receptors in endothelial cells [25]. In VEGF-activated human intestinal microvascular endothelial cells, curcumin inhibits expression of COX-2 and blocks MAPK signaling pathway [24]. Curcumin is also found to inhibit the binding of PDGF and PDGFR and reduce PDGF-stimulated phosphorylation of PDGFR [38]. However, few insights have been put on VEGF-VEGFR2 signaling pathway. Here, we reported, for the first time, that curcumin inhibited VEGF-stimulated phosphorylation of VEGFR2, which is necessary for activation of VEGFR2 during the process of HUVEC proliferation, migration and differentiation. Decrease of phosphorylation of Erk and Akt indicates that curcumin inhibited VEGFR2 mediated MAPK/Erk and PI3K-Akt pathway in endothelial cells, the former is consistent with previous study [24]. Based on these results, we confirm that curcumin can target VEGF-VEGFR2 signaling pathway and block functions mediated by VEGF. And inhibition on proliferation and migration of HUVEC induced by VEGF support our conclusion.

Apoptosis of endothelial cells plays a key role in periodic remodeling of the vasculature and in the timely evolution and regression of angiogenic response [39]. We observed that curcumin induced apoptosis of HUVEC through activating caspase-3, and it was consistent with previous studies [25]. These findings indicate that curcumin has the possibility to remodel or normalize the abnormal vasculature, and it can be indirectly confirmed by normalization of dilated sinusoidal vessels in livers of tumor-bearing mice after curcumin treatment.

Curcumin inhibits different types of tumor growth through selectively modulating multiple cell signaling pathways linked to inflammation and to survival, growth, invasion, angiogenesis and metastasis of cancer cells [18]. In our VEGF tumor model, VEGF-VEGFR2 signaling pathway plays the primary role in accelerating the tumor growth through stimulating tumor angiogenesis. However, curcumin was observed to suppress tumor size and growth in this VEGF over-expressing tumor model, and circulating VEGF and tumor microvessel density were significantly reduced after curcumin treatment. These findings reinforce that curcumin inhibits tumor growth and vasculogenesis *in vivo* through interrupting VEGF-VEGFR2 signaling pathways.

VEGF induced cancer associated systemic syndrome (CASS), including defective hematopoiesis, endocrine system, ascites, GI track disorders, muscular and adipose atrophy, and functional impairment of liver, spleen, and kidney in tumor-bearing animals and cancer patients, which impairs quality of life and shortens the lifespan [32]. VEGFR2 mediated signaling is crucial for causing the systemic damage of multiple tissues and organs [32–33]. Anti-VEGF or anti-VEGFR2 therapy significantly improves pathological changes induced by tumor-derived VEGF and confers survival advantages to tumor-bearing mice or cancer patients [14, 32, 40–41]. As a regulator of VEGF and its downstream signaling pathways, curcumin has the potential to improve paraneoplastic syndromes caused by tumor-derived VEGF. Our present study found that curcumin significantly improved severe anemia and extramedullary hematopoiesis in livers and spleens of tumor-bearing mice induced by tumor-derived VEGF and prolonged survival time of tumor-bearing mice, which provides the robust evidences that curcumin has the ability to improve quality of life of tumor-bearing mice through inhibiting VEGF mediated angiogenesis. Interestingly, low dose of curcumin used in this study showed no significant effect on tumor growth inhibition, but significantly improved anemia and extramedullary hematopoiesis in livers and spleens of tumor-bearing mice induced by VEGF, which indicates that curcumin does not always inhibit tumor size and growth, but still can benefit tumor-bearing mice through inducing systemic alterations of the vasculature that modulate the function of various tissues and organs in tumor-bearing mice.

Chemotherapy, considered as the first line regimen for cancer therapy in clinical, displays broad adverse effects [42]. Tumor-derived VEGF is found to increase chemotoxicity and chemotherapy-induced mortality through synergistically suppressing bone marrow hematopoiesis with chemotherapeutic drugs, antiangiogenic agents improve survival in tumor-bearing mice by increasing tolerance to chemotherapy-induced toxicity [34]. Curcumin, as a blocker of VEGF-VEGFR2 signaling pathway, improves defective hematopoiesis induced by VEGF, which indicates that curcumin has the possibility to decrease chemotherapy-induced toxicity. Since curcumin in combination with chemotherapeutic agents shows synergistic effect on tumor inhibition [43–44], we suppose that curcumin-based therapies may have great potential in cancer treatment. Further preclinical and clinical studies are necessary to confirm our hypothesis.

Various animal models or human studies have proved that curcumin is extremely safe even at very high doses [45–47]. A daily oral dose of 3.6 g of curcumin shows no toxicity in human and is advocated for phase II evaluation in the prevention or treatment of cancers [48]. In spite of its efficacy and safety, curcumin has not yet been approved as a therapeutic agent due to its poor bioavailability, some of possible ways including adjuvants, nanoparticles, liposomes, micelles, phospholipid complexes, derivatives and analogues are explored to overcome this problem [49]. Intraperitoneal administration is used in this study due to the poor bioavailability of curcumin, but the dose of curcumin used in this study is lower than human tolerant dose, and no difference of bodyweights between treated mice and normal mice confirms the safety of curcumin in our study.

According to our study, curcumin inhibits tumor growth, tumor angiogenesis and induces apoptosis of endothelial cells, and suppresses HIF-1 mediated VEGF production and VEGF mediated signaling pathways. The complicated mechanism of anti-angiogenesis of curcumin can be explained by the action-reaction model described previously [50]. In this model, curcumin simultaneously plays the action role and anti-reaction role, which means that curcumin not only inhibits tumor angiogenesis, but also suppresses the production of VEGF in response to hypoxia induced by inhibition of tumor angiogenesis.

Together all, our findings provide a novel mechanistic insight to explain the benefit of curcumin in preclinical and clinical studies and suggest that combinatorial therapy of curcumin with other drugs to improve pathological disorders caused by tumor-derived VEGF or chemotherapy will produce incredible benefits for cancer patients.

## MATERIALS AND METHODS

### Cell lines, cell culture

T241-VEGF, a murine fibrosarcoma T241 cell line over-expressing human VEGF, T241-vector, a murine fibrosarcoma T241 cell line transfected with the empty vector, and porcine aortic endothelial cell line over-expressing human VEGFR2 (PAE-KDR) were kindly provided by Professor Yihai Cao, Karolinska Institute, Sweden. Human umbilical vein endothelial cell (HUVEC) was purchased from American Type Culture Collection.

T241-VEGF, T241-vector and PAE-KDR were cultured in Dulbecco's modified Eagle's medium (DMEM) supplemented with 10% fetal bovine serum (FBS). HUVEC was grown in endothelial cell medium (ECM; ScienCell; 1001) supplemented with 5% FBS and 1% endothelial cell growth supplement (ECGS; ScienCell; 1052). All cell lines were incubated at 37°C under 5% CO_2_.

### VEGF stimulated HUVEC proliferation assay

1 × 10^4^ endothelial cells in 50 μL ECM medium supplemented with 0.5% FBS were seeded on opaque-walled 96-well plates and incubated overnight. Then added with various dose of curcumin (Sigma; C1386) containing 50 ng/mL VEGF (PrimeGene, China; 105–05) and incubated for 72 hours. CellTiter-Glo^®^ Luminescent Cell Viability Assay (Promega, USA; G7571) was used to determined cell viabilities.

### Cell viability assay

5 × 10^3^ cells in 50 μL medium per well were seeded on 96 well plate and incubated at 37°C overnight, then treated with 50 μL various doses of curcumin for 72 hours. 10 μL MTT solution (5 mg/mL) per well was added and incubated for 4 hours at 37°C, and then 100 μL lysis buffer (10% SDS in 10 mM HCl) was added into each well and incubated at 37°C overnight. Absorbance at 570 nm was measured.

### Endothelial cells migration assay

Endothelial cells migration was measured as described previously [51]. Briefly, cells were cultured in 6 well plates and grown to nearly confluent, then carefully scratched using a 200 μL pipette tip to draw a straight “wound” in each well. Wash twice with PBS and add different doses of curcumin, then incubated and photographed immediately at different time points. Use software ImageJ (NIH) to calculate the area percent of the “wound”, migration ratio was determined by the change of area percent of the “wound”.

### Apoptosis analysis

Cells were collected after treated with curcumin for 24 hours and washed twice with PBS, then stained with Annexin V-FITC/PI kit (Life technologies; V13242) according to the manufacture's instruction and analyzed by flow cytometry, ten thousand cells were counted.

Caspase 3/7 activities were determined by Caspase-Glo^®^ 3/7 assay (Promega, USA; G8093) according to the manufacture's instruction. Briefly, 5 × 10^3^ cells per well were seeded on opaque-walled 96-well plates and incubated at 37°C overnight, then treated with various doses of curcumin for 12 hours or 24 hours. The equal volume of caspase-Glo^®^ reagent was added into each well, incubated 30 minutes at room temperature and measured the luminescence in a plate-reading luminometer (Infinite M200, TECAN, Swiss).

### Western blot analysis

Total proteins were extracted from cells and separated using 10% SDS-PAGE and then electrophoretically transferred to a nitrocellulose membrane (Millipore; HATF00010). The membrane was blocked with 5% milk in PBST and incubated with primary antibody and secondary antibody. Target proteins were detected with ECL detection reagent (Thermo Scientific; 34075). GAPDH (Santa Cruz Biotechnology; sc-166574) and H3 (Sunshine Biotechnology, China; SAP5616) were served as reference control. Erk (9102), phospho-Erk (9101), Akt (9272), phospho-Akt (9271), FAK (3285), phospho-FAK (3283), PARP (9542) and Caspase-3 (9662) antibodies were obtained from Cell Signaling Technology.

### Detection of phosphorylation of VEGF receptor-2

Phosphorylation of VEGFR2 was investigated according to previously described [52]. Briefly, PAE-KDR cells were grown to 70–80% confluency in 6 well plates, starved for 16 hours in serum-free DMEM, and treated with various doses of curcumin for 8 hours, and stimulated with 100 ng/mL VEGF for 15 minutes. Cells were washed once with cold PBS and lysed on ice in 200 μL RIPA lysis solution supplemented with 1mM PMSF. Proteins were blotted onto nitrocellulose membranes and blocked overnight in 1% BSA in PBST, then incubated with a primary monoclonal mouse antibody against phosphotyrosine (Millipore; 05–321). Proteins were visualized using goat anti-mouse secondary antibody conjugated to HRP (Jackson ImmunoResearch; 715–035-150) and ECL detection system.

### VEGF over-expressing tumor-bearing mice model

Animal model was constructed as described previously [16, 32]. Briefly, 1 × 10^6^ tumor cells were subcutaneously implanted on the back of female 6–8 week-old C57BL/6 mice. The T241-VEGF tumor-bearing mice were randomly divided into 4 groups (*n* = 8–10/group): Control group, Curcumin (L) group, Curcumin (M) group and Curcumin (H) group. Various doses of curcumin were used for treatment according to previous studies [25–26]. Curcumin was dissolved in DMSO to a final concentration of 1 M and diluted with 20% Tween 80 in PBS. Curcumin (L) group, Curcumin (M) group and Curcumin (H) group were administered with 2.5 mM, 5 mM and 10 mM of curcumin solution, respectively (100 μL per mouse, i.p. (intraperitoneal injection), daily). Control group was treated with vehicle (20% Tween 80 in PBS, i.p., daily). All treatments started 24 hours after tumor cells inoculation and continued for 2 weeks. Tumor volumes were measured every other day and all mice were sacrificed at day 15, blood and tissues / organs were collected for further analysis.

### Blood sample analysis

Animal blood was collected by eyeball removal in the presence of anticoagulant EDTA. Hematological parameters including haemoglobin (HGB), hematocrit (HCT), red blood cells (RBC), and platelet counts (PLT) were measured by haematology analyser (Sysmex XT-2000iv, Sysmex, Japan).

### Measurement of human VEGF

The 96 well strip plate was coated with rabbit anti human VEGF monoclonal antibody (provided by Epitomics, Hangzhou, China) and incubated at 4°C overnight, blocked with blocking buffer (10 mM phosphate-buffer containing 1% BSA) at 25°C for 60 minutes, and then washed twice with washing buffer (10 mM phosphate-buffer containing 0.05% Tween-20), added 100 μL of recombinant human VEGF (R&D systems; 293-VE-50), negative control or samples and incubated at 25°C for 2 hours. Washed three times with washing buffer and incubated with avastin labeled with biotin (provided by Simcere, China) at 25°C for 60 minutes, added streptavidin coupled with horse radish peroxidase (Jackson ImmunoResearch; 016–030-084) to each well after three washes and incubated for 60 minutes at 25°C, then washed three times and added 100 μL 1-step ultra TMB-ELISA (Thermo Scientific; 34028) to each well, incubated for 15 minutes at room temperature and stopped with 50 μL 1M H_2_SO4. Measure the absorbance at 450 nm with microplate reader (Sunrise, TECAN, Swiss).

### Histological studies and Immunohistochemical examination

Paraffin-embedded tissues were sectioned in 4 μm thickness and stained with hematoxylin-eosn (H&E) according to previously described methods [16].

Immunohistochemical examination was conducted as previously described [26]. Briefly, paraffin-embedded tissue was sectioned at 4 μm, deparaffinized in xylene, and hydrated by ethyl alcohol, then antigen retrieval was performed according to citrate-EDTA antigen retrieval protocol [53]. Sections were stained with primary antibody rabbit anti mouse CD31 antibody (1:50; Abcam; ab28364) overnight, after rigorous wash with PBS for 3 times, sections were incubated with secondary antibody Goat anti Rabbit IgG-HRP (1:1000; Jackson ImmunoResearch; 415–035-166) at room temperature for 1 hour. Coloring was performed with 3, 3′-diaminobenzidine substrate-chromogen (DAB) and Mayer's hematoxylin was used for counter staining.

### Statistical analysis

GraphPad prism 5.0 was used for statistical analysis. Data was summarized as mean ± SEM. One way ANOVA was used to determine the significant differences between groups. Results were considered to be significant for *p*-values of < 0.05.
